# Smallholder poultry production in the context of increasing global food prices: roles in poverty reduction and food security

**DOI:** 10.1093/af/vfac069

**Published:** 2023-02-23

**Authors:** Mulugeta Y Birhanu, Richard Osei-Amponsah, Frederick Yeboah Obese, Tadelle Dessie

**Affiliations:** International Livestock Research Institute (ILRI), Livestock Genetics, Addis Ababa, Ethiopia; Department of Animal Science, School of Agriculture, University of Ghana, Legon, Ghana; Department of Animal Science, School of Agriculture, University of Ghana, Legon, Ghana; International Livestock Research Institute (ILRI), Livestock Genetics, Addis Ababa, Ethiopia

**Keywords:** food price, food security, poultry, poverty, smallholder

**Implications**

- A global increase in food prices severely affects the real incomes and food and nutritional security of resource-poor rural and peri-urban households in the developing world.
- During a surge in food prices, smallholder poultry production plays a significant role in poverty reduction by improving resource-poor households’ income and enhancing household food security through nutrient-rich animal source foods (ASFs) consumption. Improving its production and productivity heightens resource-poor households’ resilience to the price shock.
- Transforming existing low-input low-output smallholder poultry production systems demands technological advances in genetics, feed supply, health services, housing, and public-private partnership to deliver integrated innovation packages.

## Introduction

Global food price increases affect households’ livelihood in developed and developing economies. It impinges on the socio-economic and food and nutritional security of nations, households, and individuals within households. At the national level, the effect may include a change in the overall socio-economic and political conditions through its consequence on the trade balance and change in the commodity and labor markets. At the household and individual levels, it affects the income and expenditure of households and the nutrition, health, and education of individuals within households (Benson et al., 2008). The potential impact of increasing food prices on households’ welfare, mainly on poverty and food security, has been an interesting topic in the research and development community (Benson et al., 2008; Attanasio et al., 2013; Amolegbe et al., 2021). However, existing empirical evidence is mixed and usually debatable (Swinnen and Squicciarini, 2012). For instance, some researchers argue that increasing food prices reduces poverty (Headey, 2018). These researchers associate the effect of food price increases on increasing supply and wage responses, as most of the world’s poor livelihood is strongly associated with agriculture. Other researchers suggest that rising food prices would increase poverty, mainly in urban areas and among poor rural households, as most resource-poor households are usually net buyers of different food groups (Ivanic andMartin, 2008; Hoyos and Medvedev, 2011). For some researchers, increasing food prices have temporal effects. Accordingly, rising food prices increase poverty in the short run, as most poor households spend their budget on food. It reduces poverty in the long run by increasing smallholder producers’ wage rates and profits (Ivanic and Martin, 2014). Nevertheless, conclusions from most empirical findings rely on various assumptions, and generalizing most results to the heterogeneous global population seems highly unlikely.

A change in food price is usually a signal of possible changes in households’ food security in the developing world (Kalkuhl et al., 2016). Food security has various dimensions related to availability (total supply), accessibility (affected by real income), utilization (daily dietary needs), and stability (temporal condition). An increasing food price may affect either of these dimensions, such as accessibility, by reducing the real income of households or their utilization. Like the poverty impacts, an increase in food price may have a mixed effect on households’ food security. Most researchers like Sophal (2011) examined its adverse effects, and others such as Amolegbe et al. (2021) documented both negative and positive impacts based on the origin of foods produced (imported vs. domestic). Despite mixed evidence on the welfare impact of an increase in food prices, most studies agree that resource-poor households are the primary victims of price increases, at least in the short run (Attanasio et al., 2013). An increasing food price may lead the poor to hunger as they are forced to buy a low basket of foods with their limited income.

Existing studies show that an increasing food price can be a challenge and/or opportunity for smallholder farmers in developing countries (Hella et al., 2011). The effect may depend on households’ status in the food system and whether they are net food buyers or sellers. Usually, smallholder producers who sell most of their agricultural products can benefit more from the increase in price than net buyers. However, as indicated by Nakelsea et al. (2018), the status of households as net buyers or net sellers may not be a sufficient condition, and other factors can influence the overall effect. For instance, Benson et al. (2008) noted that increasing food prices could create a positive opportunity for farmers by persuading them to produce more if there are no input and output market access constraints.

Studies on food price increases mainly focus on staple food crops such as maize, wheat, and rice; empirical evidence on livestock products, specifically poultry products, is very limited. Therefore, in this paper, we examined the potential role of smallholder poultry production in reducing poverty and enhancing food security among resource-poor households, mainly during food price increases. We used Rural Household Multiple Indicator Survey (RHoMIS) datasets that contain multiple indicators collected from 2015 to 2020 in 33 countries and the FAO databases (RHoMIS, 2021; FAO, 2022) to generate some empirical evidence. We used the data from 24 (Countries included are Burkina Faso, Burundi, Cambodia, Comoros, Cote d’ivoire, DRC, El Salvador, Ethiopia, Ghana, Honduras, Kenya, Lao PDR, Malawi, Mali, Nicaragua, Niger, Nigeria, Rwanda, Senegal, Sierra Leone, Tanzania, Uganda, Vietnam, and Zambia.) countries (31,484 households) by excluding 9 countries in the Arab States, South Asia, and countries categorized as upper middle-income countries. Countries in the RHoMIS datasets are the foundation for generating indicators from the FAO datasets. We generated data on regional-level smallholders’ livestock holding, the contribution of poultry production to household cash income, the global trend in food prices, regional supply and consumption of animal source food and the status of moderate or severe food insecurity in developing and developed countries. This paper highlights the scope of smallholder poultry production in the developing world, trends in global food price increases, the role of smallholder poultry production in poverty reduction and food security, opportunities for improved poultry production and productivity, and provides conclusion and policy implications for the future.

## Smallholder Poultry Production in the Developing World

Poultry production is an integral part of smallholder agriculture in the developing world and has a multidimensional contribution to the livelihood of both rural and urban households. Its contribution spans economic and social to cultural and environmental benefits (Gueye, 2000; Akinola and Essien, 2011; Birhanu et al., 2022). The economic contributions mainly include its role in generating additional income for the family that can be used to pay school fees, cover medical expenses, and even buy other agricultural inputs such as fertilizer and improved seeds (Birhanu et al., 2021b). Income from poultry production is generated from the sale of eggs, meat, live chickens, and sometimes the sale of manure. Smallholder poultry production is households’ primary source of high-quality protein (Wong et al., 2017). Also, the birds are raised for other socio-cultural purposes, such as religious ceremonies and festivals, gifts, and cock fighting (Birhanu et al., 2022). The birds can also serve as an asset that can be quickly converted into cash during emergencies or unexpected household expenses.

## Scope and status of smallholder poultry production

**Figure 1.**
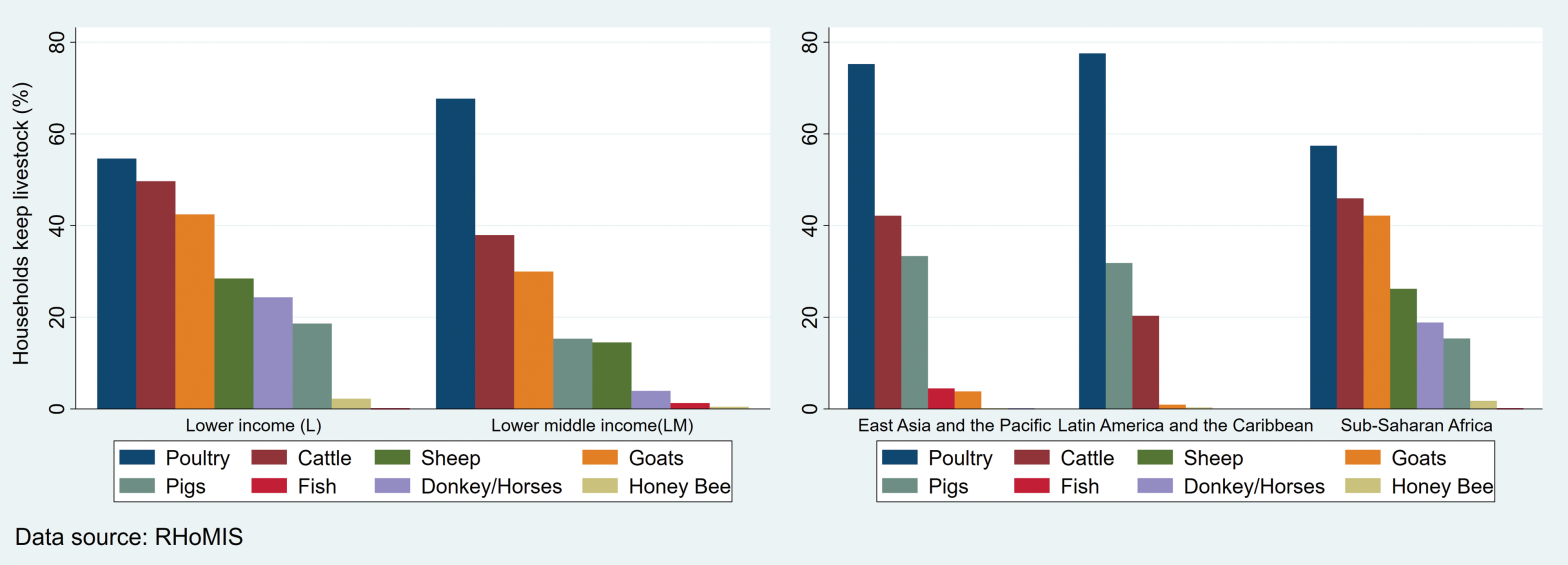
Proportion of smallholder farmers that kept different livestock species in developing regions (2015 to 2020).

Rural households in developing countries are very heterogeneous. They vary, including resource endowments, land holdings, livestock ownership, and socio-cultural and environmental aspects. In most developing countries, livestock holding is one of the indicators of household socio-economic status, as livestock are the primary sources of household food and income. Among others, poultry is the primary livestock species kept by smallholder farmers, and it is a vital livestock production activity in rural and peri-urban areas. Figure 1 presents the proportion of sampled households keeping different livestock species from 2015 to 2020 in 24 developing countries disaggregated by the World Bank income classification of countries and developing regions. In the lower-income countries, 55% of the households kept poultry (mainly chicken), followed by cattle (50%) and goats (42%). A total of 68% of households in the lower-middle-income countries also kept poultry, followed by cattle (38%) and goats (30%). When we disaggregated by developing sub-regions, a higher proportion of households in Latin America, the Caribbean, East Asia, and the Pacific kept poultry than those in Sub-Saharan Africa (SSA). Poultry was owned by 75%, 78%, and 57% of the sampled rural households in East Asia and the Pacific, Latin America and the Caribbean, and Sub-Saharan Africa, respectively. Poultry was kept by most rural households, which may indicate the vital role of poultry production in rural households’ livelihoods.

## Trends in global food prices and the price of poultry products

**Figure 2.**
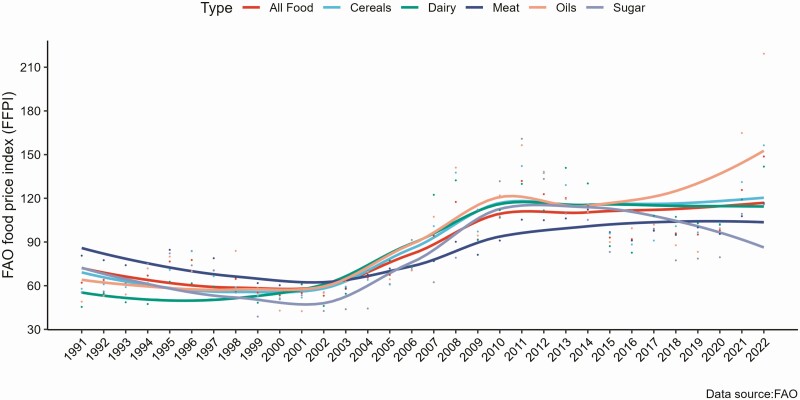
FAO Food Price Index (FFPI) trends in the previous three decades (1991 to 2022).

An increasing trend in food prices has become the primary global social and economic concern in the previous few decades as it makes the cost of basic household food more expensive for resource-poor households. It affects both the quantity and quality of foods consumed by households. Evidence from the FAO global food price change indicates that food prices showed an overall increasing trend in the previous three decades (1991 to 2022), including the price of meat and dairy products (Figure 2). For instance, in 2021, the average food price index was 125.7, 27.6 points greater than the index in 2020. Similarly, the price index for meat was 107.7, which is 12.2 points greater than the year before. Among the five food groups, the oil food groups experienced the highest increase in price, which showed 65.4 points increment in 2021 than the previous year. These may include some feed ingredients used in the commercial poultry production sector, which has implications on the price of eggs and poultry meat.

**Figure 3.**
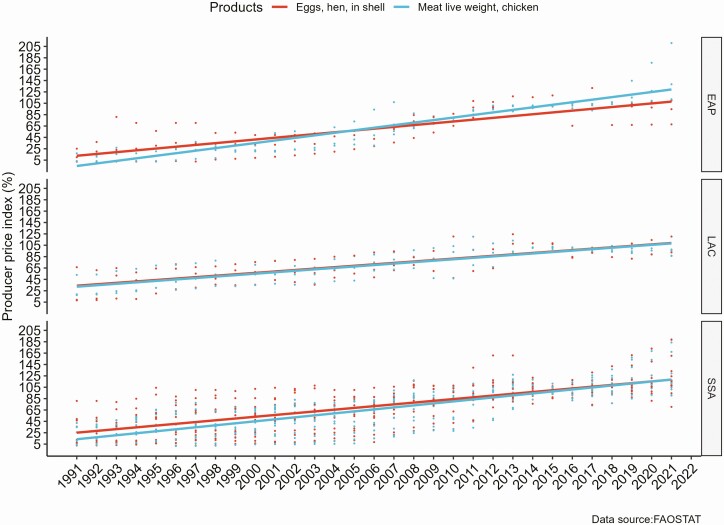
Producer Price Index (PPI) for eggs and chicken meat in developing sub-regions (2014 to 2016 = 100).

Like other food products, there was an increasing trend in the price of eggs and chicken in the previous three decades. Figure 3 shows the producer price index (PPI) for eggs and chicken from 1991 to 2022, mainly in countries where the RHoMIS data was collected. The PPI shows domestic producers’ average prices of eggs and chicken at the farm gate, which indicates the change in the prices of products from the producers’ perspective. There was a significant increase in the prices of eggs and chicken in the three developing sub-regions, East Asia and the Pacific (EAP), Latin America and the Caribbean (LAC), and SSA. The increased producers’ price would increase the net income generated from poultry production as most smallholder poultry producers use limited purchased inputs for production.

## Role of smallholder poultry production in  poverty reduction

According to the new global poverty line, under 700-million people live in extreme poverty. Most of these people reside in low- and middle-income countries, mainly in Africa and Asia. The recent Covid-19 pandemic and increasing price of oil, fertilizers, and other essential food items resulting from the Russian–Ukraine crisis also potentially increased poverty in developing regions. Rising prices would heighten the poverty incidence and mainly affect the poor by increasing the cost of living and making most nonpoor people enter poverty (Easterly and Fischer, 2001) or pushing back people who recently escaped poverty. Therefore, there is a need to develop strategies that will enhance the resilience of the most vulnerable households in developing regions.

One of the strategies suggested is creating jobs and employment opportunities that enhance the income of resource-poor households (Ayoo, 2021). In the rural areas of developing countries, households’ livelihood mainly depends on agricultural activities, including crop and livestock production and wages earned from the farm and nonfarm activities. Although most rural households are subsistence-based, they usually generate income by selling surplus crops and livestock products to cover other expenses. In this regard, poultry production can play an important role, as it is most rural households’ primary livestock production activity. Moreover, since women play major roles in poultry production, poultry production helps to reduce inequality by enhancing their income and control over resources.

The potential contribution of poultry production to households’ income may vary from household to household, depending on the households’ socio-economic status and other cultural factors. As the dominant livestock are kept by resource-poor households, the relative income contribution for these households would be higher than resource-rich households. We used the RHoMIS data to examine the contribution of poultry cash income to livestock and household cash incomes from 2015 to 2020. In this case, the poultry cash income constitutes income from selling poultry (chicken, duck, and other poultry) and poultry products (egg/meat/live chicken). The livestock cash income refers to income from selling livestock and livestock products, including cattle, sheep, goats, horses/donkeys, pigs, chickens, ducks, and other poultry. Likewise, household cash income refers to the income from the sale of livestock and livestock products, the sale of crop and crop products, and income generated from off-farm activities. We examined the proportion of poultry cash income by disaggregating it with the overall household income quintile. Unlike the cash income indicators, the overall household income quintile was constructed from cash and noncash incomes estimated from on-farm and off-farm livelihood activities, including the value of owned crops and livestock products consumed at home. The first- and second-income quintiles (Q1 and Q2) can represent households with lower income or poor households, and the fourth- and fifth-income quintiles (Q4 and Q5) can represent households with higher income or rich households.

**Figure 4.**
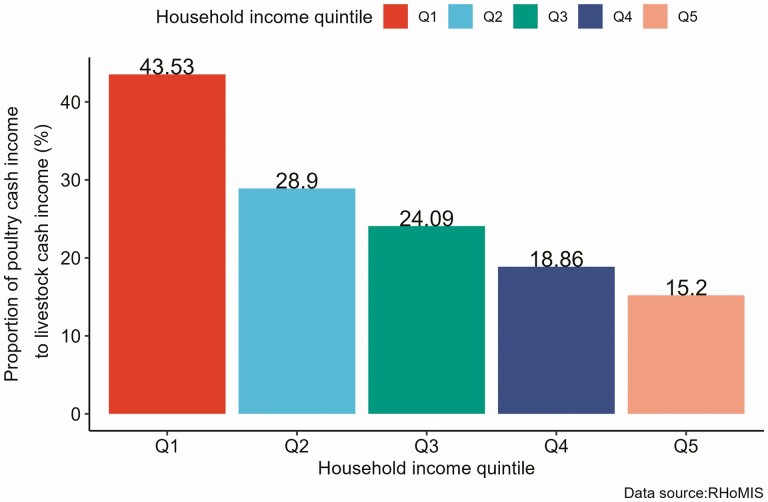
Average contribution (%) of poultry cash income to livestock cash income (2015 to 2020).

**Figure 5.**
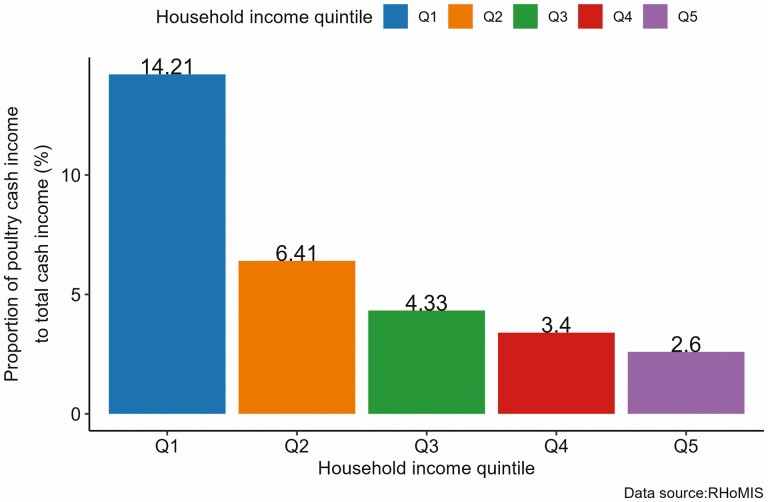
Average contribution (%) of poultry cash income to household cash income (2015 to 2020).

Results show that the proportion of cash income generated from the poultry production was higher for the poorest households than for the richest households (Figures 4 and 5). When the proportion of poultry cash income from livestock cash income was considered, households in the first quintile (Q1), the poorest 20% of households, generated the highest (43.5%) cash income proportion, followed by households in the second quintile (Q2), 28.9% (Figure 4). In comparison, the top 20% of richest households (Q5) generated 15.2% of the livestock cash income from poultry production. For some households, cash income from poultry production was the only reported livestock cash income; most were found in the lowest 40% (Q1 and Q2) households. There was also significant variability in the average poultry cash income contribution from country to country, ranging from 2.11% to 85.25%, depending on the country’s socio-economic status.

Likewise, the contribution of poultry cash income to household cash income was significantly higher for households in the lower-income quintile than households in the higher-income quintile. Poultry cash income accounted for 14.2% of households’ cash income in the lowest 20% of poor households (Q1), while it accounted for 2.6% of the top 20% or the richest households (Q5; Figure 5). This could be associated with fewer livelihood opportunities for the poorest households, affecting their potential to generate more income. Although the average contribution of poultry cash income to overall household cash income seems low, it has significant implications for households living below the poverty line. The higher contribution of poultry cash income to households’ livestock and total cash income in the first and second quintiles (Q1 and Q2) may suggest poultry production is one of the major livelihood activities for resource-poor households and its potential role as an entry point for poverty reduction and enhancing households’ livelihood.

Since most resource-poor households are net sellers of poultry products, the income contribution increases when the price of eggs and live birds/meat increases, especially during foods’ price increases. This can be further enhanced by improving the production and productivity of the sector through integrated intervention packages and propoor development strategies. The intervention packages may include locally adapted and farmer-preferred improved breeds, best-cost feeds, vaccines and treatment drugs, improved housing, technical and entrepreneurial capacity buildings, and tailored credit and marketing services.

Compared to other livestock and crop-related interventions, poverty reduction strategies based on smallholder poultry production may have a comparative advantage during food price increases. Firstly, an increasing price coupled with a rising trend in animal-source food consumption, mainly white meat and eggs, would create better market opportunities for smallholder farmers, enhancing income generation opportunities. Secondly, for most poor rural households’, poultry production is an integral part of their livelihood, and they have adequate management experience. This increases the success of interventions. Thirdly, poultry production at the smallholder level has very low startup and running costs that are not significantly affected by the global price increase. Locally adapted birds can easily survive by scavenging around the home, with limited supplementary feeds obtained from locally available sources and household wastes. Hence an increase in the prices of eggs and chicken may generate an additional gain in income. Fourthly, recent advances in improved poultry genetics have brought locally adapted and farmer-preferred dual-purpose breeds that can significantly increase the production and productivity of smallholder farmers within a very short period. Unlike indigenous breeds that take more than 24 wk to reach slaughter weight, some dual-purpose breeds can achieve higher body weight in 12 wk. This helps smallholders to produce live chicken/meat within short periods and mitigate the adverse income effects of food price increases in the short run. Experience from Tropical Poultry Genetics Solution (TPGS) project activities in Africa shows that tropically adapted dual-purpose improved chicken breeds-based production generates a significant gain in income compared to indigenous breeds-based production (Birhanu et al., 2022). Similarly, lessons from other poultry development projects in developing countries show the potential contribution of adopting such improved breeds to increase household income (Islam and Jabbar, 2005; Kumaresan et al., 2008).

## Role of Smallholder Poultry Production in Food Security

Food insecurity has been a global development challenge in previous decades. In 2020, between 720- and 811-million people experienced hunger globally (FAO et al., 2021). About 418 million (more than half) in Asia and 218 million (more than one-third) in Africa were affected by hunger in 2020. Most of the sub-regions in Africa (Middle and Eastern Africa), Asia (Southern and Western Asia) and America (Central America) and the Caribbean were highly affected by undernourishment. In most developing economies, undernourishment is strongly associated with limited animal source foods (ASFs) consumption (Adesogan et al., 2020). Due to its effect on the quantity and quality of food consumed, the existing challenge would be exacerbated during global and local increases in food prices.

**Figure 6.**
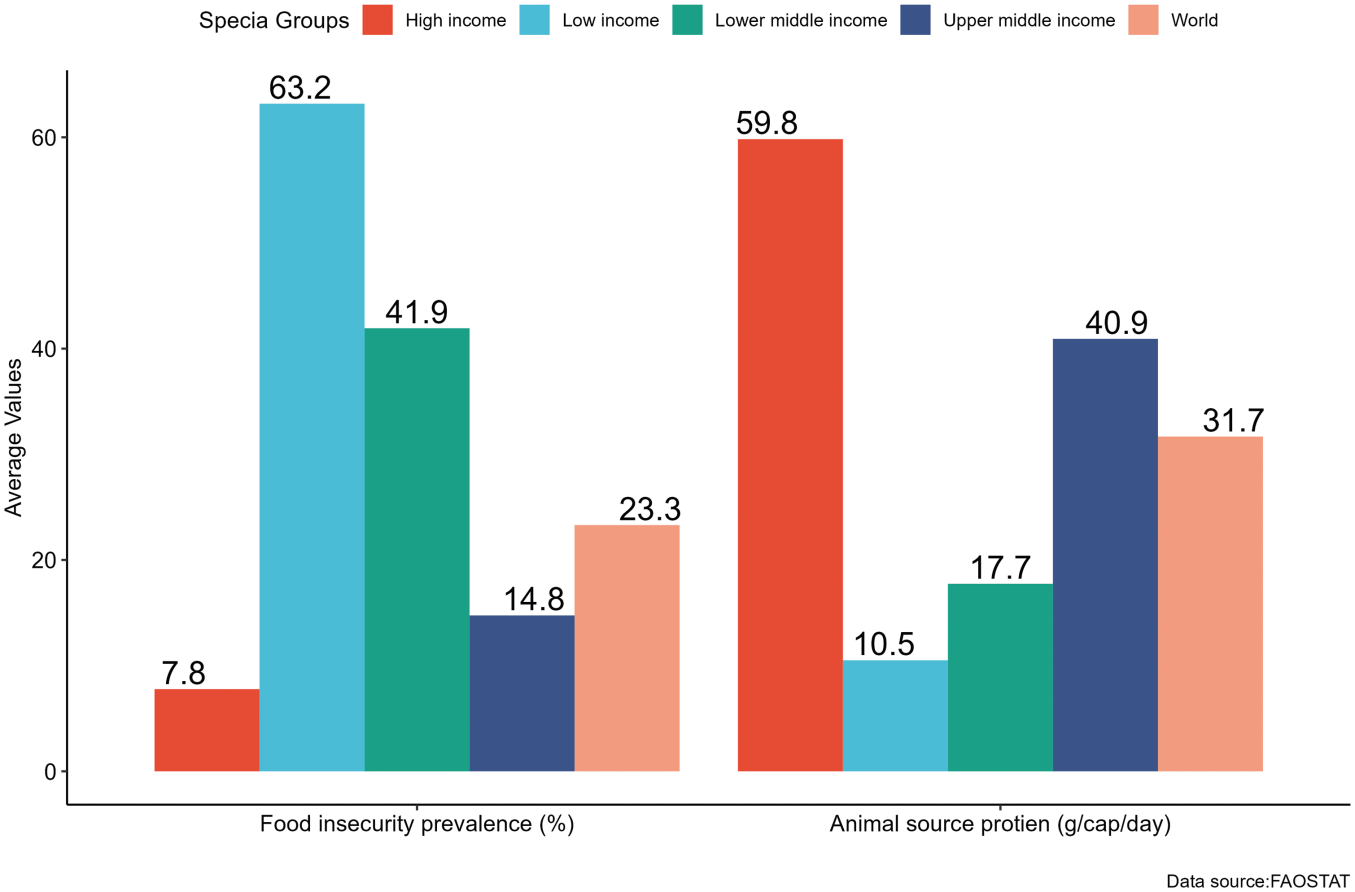
Average food insecurity prevalence (%) and animal-source protein supply (g/cap/day) by income classification (2014 to 2019).

Historical data from 2014 to 2019 on countries’ food supply and food security status indicate an inverse association between animal sources’ food supply and moderate and severe food insecurity in the nations (Figure 6). Developing countries are characterized by a higher prevalence of food insecurity and a limited supply of ASFs. For instance, the average per capita supply of animal source protein in lower-income and lower-middle-income countries (The average for lower-income, lower-middle-income, and upper-middle-incomecountries are based on countries in the RHoMIS datasets. Higher-incomeand World averages are global indicators.) were significantly lower than that in high-income and upper-middle-income countries. The 3-year averages (2014 to 2016, 2015 to 2017, 2016 to 2018, 2017 to 2019) for animal-source protein supply and prevalence of moderate and severe food insecurity in lower-income countries were 10.5 (g/cap/day) and 63.2%, respectively. However, the three-year average for animal-source protein supply in high-income countries was 59.8 (g/cap/day). At the same time, moderate and severe food insecurity prevalence was 7.8%. This demonstrates the strong association between animal-source protein supply and the food security status of countries globally and suggests increasing the supply of ASFs, including eggs and poultry meat, to enhance the food security status of households in developing countries.

In most developing countries, the average price of ASFs is much higher than crop-based staple foods. The increasing global food price generally makes households’ daily food baskets more expensive, and resource-poor households adopt various strategies to mitigate the rising price effect. This may include replacing expensive foods (i.e., meat and eggs) with cheaper alternatives, stopping consuming other foods or reducing the quantity and quality of food consumed (Mkhawani et al., 2016), which affects the food and nutritional security of households. Smallholder poultry production can play a significant role in this situation by directly supplying cheap ASFs (eggs and meat) to vulnerable households (Wong et al., 2017; Daghir et al., 2021) or by generating additional income to purchase other food items.

Poultry meat and eggs are the most crucial sources of proteins and other essential nutrients. Poultry meat has low fats and cholesterol, is rich in n-3 polyunsaturated fatty acids and contains highly digestible protein, B-group vitamins (mainly thiamin, vitamin B6, and pantothenic acid), and various minerals (like iron, zinc, and copper; Bordoni and Danesi, 2017). Similarly, poultry egg is rich in protein, lipids, vitamins, Choline, minerals, and trace elements (Réhault-Godbert et al., 2019). Accessibility to resource-poor households and multiple nutritional values makes poultry products the most preferred ASFs in the developing world. Households can easily access poultry products by raising birds in their backyard at a lower cost than other animal source proteins. Thus, improving the sector’s production and productivity and enhancing households’ awareness of knowledge-based consumption will help to reduce the adverse food and nutritional security effect of food price increases.

## Exploring Opportunities for Improved Poultry Production and Productivity

Although smallholder poultry production has an inimitable potential contribution to poverty reduction and improving food security in developing countries, it is characterized by low production and productivity and huge gaps between potential and actual productivities (Birhanu et al., 2021a). This could mainly be associated with the low genetic potential of existing chicken breeds, a traditional and low input management system, inadequate access to inputs and market outlets, and limited institutional and policy support. Hence, research and development efforts that aim to improve the production and productivity of the sector can adopt multiple strategies, including the following approaches: Improving the productivity of existing chicken breeds through better management and breed improvement; introducing innovative and improved poultry packages. Existing traditional management system restricts the genetic potential of locally available breeds, and researchers suggest improving the management systems to increase the production and productivity of these breeds (Henning and Pym, 2019). Innovative packages may include delivery of locally adapted and farmer-preferred strains, delivery of best-cost feeds (affordable improved feeds) and affordable health packages, creating market linkages, introducing improved market infrastructure, and developing the value chain.

Lessons learned from the TPGS project activities in SSA indicate that introducing locally adapted and farmer-preferred dual-purpose breeds transforms the production and productivity of the sector within a short time. The project tested and delivered fully vaccinated and vigorous chicken strains using an innovative business model through public–private partnerships. The introduced breeds resulted in a significant reduction in chicken mortality and increased productivity in terms of live body weight (200% to 300%) and egg production (150% to 200%) compared to local breeds. The observed gain in production and productivity increased household income and consumption of poultry products (Birhanu et al., 2022). In addition to their adaptability in the tropics, some dual-purpose strains have good scavenging abilities that minimize feed costs and provide other environmental benefits. The above evidence demonstrates existing opportunities to improve the production and productivity of the sector and enhance the livelihood of resource-poor households, especially during an increase in food prices.

## Conclusions

Most poor households with food insecurity are found in developing countries, mainly in the rural and agricultural sectors. These households have limited resilience to local and global socio-economic and environmental shocks, including a surge in the price of foods. Increasing food price mainly reduces the real income of these households and affects the quality and quantity of the basket of foods households consume. This may push some of them to slip into poverty and food insecurity. On the other hand, increasing food prices may improve smallholder producers’ income, at least in the long run, by enhancing their profitability and creating jobs and employment opportunities. Hence, the net effect may depend on the level of agricultural production and productivity, the type and amount of food purchased from the market, and constraints that affect households’ production and market participation. In this regard, smallholder poultry production is vital in resource-poor settings as it helps households generate additional income with limited investment and provide cheap ASFs rich in protein and other nutrients. Hence, improving the production and productivity of the widely adopted traditional production system would help to mitigate the negative effect of food price shocks and contributes to poverty reduction and enhance food security. Increasing eggs and meat productivity would simultaneously boost the income and consumption of ASFs among resource-poor rural and peri-urban households.

## About the Author(s)

**Mulugeta Y. Birhanu** is a Research Officer (Socio-economist) at International Livestock Research Institute (ILRI), Addis Ababa, Ethiopia. He holds BA in Economics (Haramaya University, Ethiopia) and MSc in Research Methods (Jomo Kenyatta University of Agriculture and Technology, Kenya). He has also Diploma in General Agriculture. He worked at International Center for Agricultural Research in the Dry Areas (ICARDA) as a Research Officer in the Social, Economic and Policy Research Program. Mulugeta also worked at Haramaya University as Lecturer and Researcher in the School of Agricultural Economics and Agribusiness Management. He has adequate research experience in developing countries, mainly in Africa and Southeast Asia. His research interest includes the adoption and use of improved technologies, productivity and efficiency analysis, poverty and food security analysis, value chain analysis, sustainability, impact assessment, and business models for scaling improved agricultural technologies.

**Richard Osei-Amponsah** is an Associate Professor of Animal Science at the University of Ghana. His research focus is on utilization of quantitative and molecular genetics as tools for the sustainable management of Animal Genetic Resources (AnGR). He has expertise in development of sustainable animal breeding programmes for low input-livestock systems as well as National and Regional Strategic Action Plans for AnGR. He has contributed to capacity building efforts through student training and served as a resource person at various agricultural stakeholder training workshops. He has a number of publications on AnGR management to his credit and has actively participated in international and local conferences. He is a member of the AU-IBAR’s African Technology and Innovations incubators Advisory Committee (ATIIAC) for Africa, an Executive Committee Member of the All-African Society of Animal Production (AASAP) and the President of the Ghana Society of Animal Production (GSAP).

**Frederick Yeboah Obese** is a Professor at the Department of Animal Science, School of Agriculture, College of Basic and Applied Sciences, University of Ghana, Legon. He holds a Bachelor of Science (Hons) in Agriculture and Master of Animal Science degrees from the Kwame Nkrumah University of Science and Technology, Kumasi, Ghana, and a PhD degree in Animal Science from the University of Melbourne, Australia. His expertise is in the area of Reproductive endocrinology and physiology in livestock, development of feed packages for ruminant livestock, as well as climate change and livestock production. He has research experience working with partners from Australia, the United States, the United Kingdom, and other African countries, including Kenya and Malawi. Frederick Yeboah Obese has participated in a number of international conferences and presented papers, as well as, authored or coauthored over 100 articles in peer-reviewed journals.

**Tadelle Dessie** is Principal Scientist, Animal genetics/breeding at International Livestock Research Institute (ILRI). He is the Program Director for Tropical Poultry Genetics Solution (TPGS), implemented in Africa and Southeast Asia. He holds a BSc in Animal Science, MSc in Sustainable Tropical Animal Production Systems, and a PhD in Animal Genetics and Breeding. Tadelle has more than 25 years of research and development experience in various national and international research and development organizations. Tadelle has authored or coauthored more than 250 publications in peer-reviewed journals and is coauthor of the first chicken genome book. He is a member of several professional associations and networks and served as President of the Ethiopian Society of Animal Production (ESAP) and All African Society for Animal Production (AASAP); he is a Fellow of the Ethiopian Academy of Sciences and is also a regular reviewer for many scientific journals. He received a number of awards in recognition of his social competence and scientific achievements.
